# Efficacy and safety of vedolizumab for pediatrics with inflammatory bowel disease: a systematic review

**DOI:** 10.1186/s12887-022-03229-x

**Published:** 2022-04-04

**Authors:** Shengbo Fang, Yanqing Song, Chunyan Zhang, Libo Wang

**Affiliations:** 1 Department of Pharmacy, First Hospital of Jilin University, Changchun, China; 2 Department of Pediatric Gastroenterology Unit, First Hospital of Jilin University, Changchun, China

**Keywords:** Inflammatory bowel disease, Vedolizumab, Pediatrics, Systematic review

## Abstract

**Background:**

Vedolizumab use in pediatrics is still off-label and the data are limited. We conducted a systematic review evaluating the efficacy and safety of vedolizumab in children and adolescents with inflammatory bowel disease (IBD).

**Methods:**

PubMed, EMBASE and Cochrane databases were systematically searched for studies of vedolizumab in children and adolescents with IBD reporting clinical remission, response, corticosteroid-free (CS-free) remission, mucosal healing, or safety up to December 3^rd^ 2021.

**Results:**

Ten studies, comprising 455 patients were included. For CD, the pooled clinical remission rates were 25% (19/75) at 6 weeks, 28% (25/85) at 14 weeks, 32% (17/53) at 22 weeks, and 46% (43/92) at 1 year. For UC/IBD-U, the pooled clinical remission rates were 36% (25/70) at 6 weeks, 48% (52/101) at 14 weeks, 53% (24/45) at 22 weeks, and 45% (50/112) at 1 year. Mucosal healing was found in 17%-39% of CD and 15%-34% of UC/IBD-U respectively. Six percent of patients reported serious adverse events.

**Conclusions:**

According to low-quality evidence based on case series, approximately one-third and one-half of patients for CD and UC/IBD-U respectively achieved remission within 22 weeks, and about half of patients achieved remission at 1 year with reasonable safety profile. Long-term benefit profile data and high quality evidence are still needed.

**Supplementary Information:**

The online version contains supplementary material available at 10.1186/s12887-022-03229-x.

## Background

Medical therapies commonly used for inflammatory bowel disease (IBD) include aminosalicylates, corticosteroids, immune modifiers, biologic agents, antibiotics and probiotics [1]. As IBD relapse rate is high, some patients might become corticosteroid-dependent or corticosteroid-resistant. It is reported that the rate of steroid dependency is much higher in children than in adults (45% vs. 8% respectively) [2]. Besides, although anti-TNF agents have been a significant breakthrough in the treatment of IBD, approximately ~ 10%-40% patients do not improve after therapy (primary non-response), and ~ 20%-40% may lose response to therapy overtime (second loss of response) [3–7]. Therefore, there is still a great need for new drugs with other mechanisms of action that act on different inflammatory pathways involved in the pathogenesis of IBD [8].

Vedolizumab is a novel, fully humanized immunoglobulin G1 monoclonal antibody selective for the gut. It can block only α4β7 integrin that inhibits adhesion of a gut-homing subset of T lymphocytes to mucosal addressing cellular adhesion molecule-1 (MAdCAM-1) [9]. For adults, the efficacy and safety of vedolizumab on moderate-to-severely active UC or CD have been established by GEMINI clinical trials [10–12], and got marketing approval in May 2014 in the USA and later in Europe [13, 14]. Guidelines suggested vedolizumab could be used in the treatment of UC where anti-TNF therapy had failed [15], and UC or CD who was refractory to steroids or anti-TNF [16, 17]. For children, European Crohn's and Colitis Organization (ECCO) and European Society for Pediatric Gastroenterology Hepatology and Nutrition (ESPGHAN) recommended vedolizumab for UC in chronically active or steroid-dependent patients as second line biologic therapy after anti-TNF failure [18], and for CD in patients who fail to achieve or maintain clinical remission on anti-TNF agents, despite anti-TNF dose optimization and immunomodulator use [19]. Nevertheless, vedolizumab use in pediatric population is still off-label and the efficacy evidence is insufficient. Given that the increasing use of vedolizumab in pediatrics, safety monitoring is essential, as it is suggested that drug safety must be demonstrated independently from adult studies and couldn’t be extrapolated [20].

The aim of this study was to summarize the current evidence and to assess the efficacy and safety of vedolizumab for children and adolescents with IBD.

## Methods

The systematic review was registered in PROSPERO (registration number: CRD42020222828) and was performed in accordance with the guidelines established by the PRISMA statement [21].

### Literature search

We systematically searched PubMed, EMBASE and Cochrane Library databases from inception to December 3^rd^ 2021 using the following search terms: “inflammatory bowel disease”, “vedolizumab”, “child” and “adolescent”. The full search strategy is detailed in Supplementary Data (Table S1). Language or publication type was without restriction.

### Inclusion criteria

Studies that met the following criteria were included in this systematic review: (a) studies carried out in children and adolescents with pediatric onset (< 18 years) IBD (CD, UC, unclassified), remaining under pediatric monitoring, and evaluation up to 21 years old were included; (b) treatment with vedolizumab alone or combination with other agents; (c) studies written in English.

### Exclusion criteria

Studies were excluded according to the following criteria: (a) studies on non-human subjects; (b) studies conducted on adults subjects; (c) the number of case series is less than five; (d) studies that were letters or editorial; (e) studies that lacked sufficient raw data; (f) studies that were duplicated; (g) studies that were ongoing or not finished; (h) abstract only.

### Outcomes and endpoints

The primary outcome measure of this systematic review was clinical remission; second outcome measures included: (a) clinical response; (b) corticosteroid-free (CS-free) clinical remission; (c) mucosal healing; (d) safety (any adverse event that was judged related to vedolizumab by authors of the primary study). Clinical remission, clinical response and CS-free rates were collected after first dose where available. The definition of clinical remission, clinical response, CS-free clinical remission and mucosal healing varied in different studies and were summarized in Table 1.

**Table 1:** Characteristics of included studies

Study characteristics			Outcome definitions		
**First author[year]**	**Country**	**Design**	**Remission criteria(clinical remission or CS-free clinical remission)**	**Response criteria**	**Mucosal healing criteria**
Singh[2016] [22]	USA	Retrospective case series	Clinical remission: PUCAI < 10 or wPCDAI < 12.5	NR	NR
Conrad[2016] [23]	USA	Prospective case series	Steroid-free remission: Inactive disease by PCDAI (≤ 10) or PUCAI (< 10) and no current corticosteroid therapy	PCDAI decrease ≥ 12.5;PUCAI decrease ≥ 20;	NR
Ledder[2017] [24]	Multi-country	Retrospective case series	Steroid-and exclusive enteral nutrition [EEN]-free remission: wPCDAI < 12.5 or PUCAI < 10 without the need for new medications or surgical intervention;	NR	SES-CD < 3 in CD or UCEIS = 0 in UC/IBD-U
Schneider[2018] [25]	Austria	Retrospective case series	Clinical remission: shPCDAI < 10 points; PUCAI < 10 points	NR	NR
Olbjørn[2020] [26]	Norway	Case series	NR	NR	NR
Jossen[2020] [27]	USA	Retrospective case series	CS-free clinical remission: PCDAI < 12.5, or partial Mayo score < 2 and off corticosteroids	NR	Endoscopic remission and Histologic remission: Endoscopic remission: Mayo Score = 0;SES-CD score ≤ 2; Histological remission: Nancy Index score ≤ 1 in UC and a CD histologic activity score ≤ 1 in every segment;
Dolinger[2021] [28]	USA	Prospective case series	CS-free remission: wPCDAI ≤ 12.5 or pMS < 2, and no form of corticosteroids for at least 4 weeks	NR	NR
Fabiszewska[2021] [29]	Poland	Retrospective case series	Clinical remission: PCDAI ≤ 10 or PUCAI ≤ 10 after induction phase (4th dose week) and maintenance phase (10th dose week)	PCDAI decrease ≥ 12.5;PUCAI decrease ≥ 20 after 4th dose week	The level of FCP: a statistically significant decrease in FCP level between baseline and after vedolizumab commencement
Garcia-Romero [2021] [30]	Span	Retrospective case series	Clinical remission: PCDAI < 10 or PUCAI < 10	PCDAI decrease ≥ 15 and final PCDAI < 30;. PUCAI decrease ≥ 20	NR
Hajjat[2021] [31]	USA	Retrospective case series	CS-free clinical remission: PCDAI ≤ 10;PUCAI < 10	NR	NR

Abbreviations: *PCDAI* pediatric Crohn’s disease activity index, *wPCDAI* weighted pediatric Crohn’s disease activity index, *shPCDAI* short pediatric Crohn’s disease activity index, *PUCAI* pediatric ulcerative disease activity index, *pMS* partial Mayo score, *CS* corticosteroids, *SES-CD* simple endoscopic score for Crohn’s disease, *UCEIS* ulcerative colitis endoscopic index of severity, *CD* Crohn’s disease, *UC* ulcerative disease, *IBD-U* inflammatory bowel disease unclassified, *NR* not reported, *FCP* fecal calprotectin

### Data extraction

All the potentially related articles were retained by two authors (FSB, SYQ) independently, and the full texts were strictly reviewed according to inclusion/exclusion criteria regarding to preset outcomes. Any disagreements were resolved by consensus or consulted with a senior author (WLB). For the included studies, the following items were extracted: study characteristics (author, year of publication, country, study design), patients characteristics (age, type of IBD, disease behavior, percentage of anti-TNF experienced), vedolizumab dosage, clinical efficacy and adverse events (AEs).

### Methodological assessment

For quality assessment, a validated quality appraisal tool developed by the Canadian Institute of Health Economics (IHE) was used for case series [32], including study objectives, population, interventions and co-interventions, outcome measures, statistical analysis, results and conclusions and competing interests. A study with 14 or more yes responses (≥ 70%) was considered to be of acceptable quality [33]. The grade of evidence was showed in Table S2.

### Statistical analysis

We provided descriptive statistics. Continuous parametric data are presented as mean and standard deviation (SD), while nonparametric data presented as median followed by range or interquartile range (IQR), unless otherwise specified. The categorical data of the outcome measures are expressed as percentage of total cases with 95% confidence interval (95% CI).

## Results

**Fig. 1:**
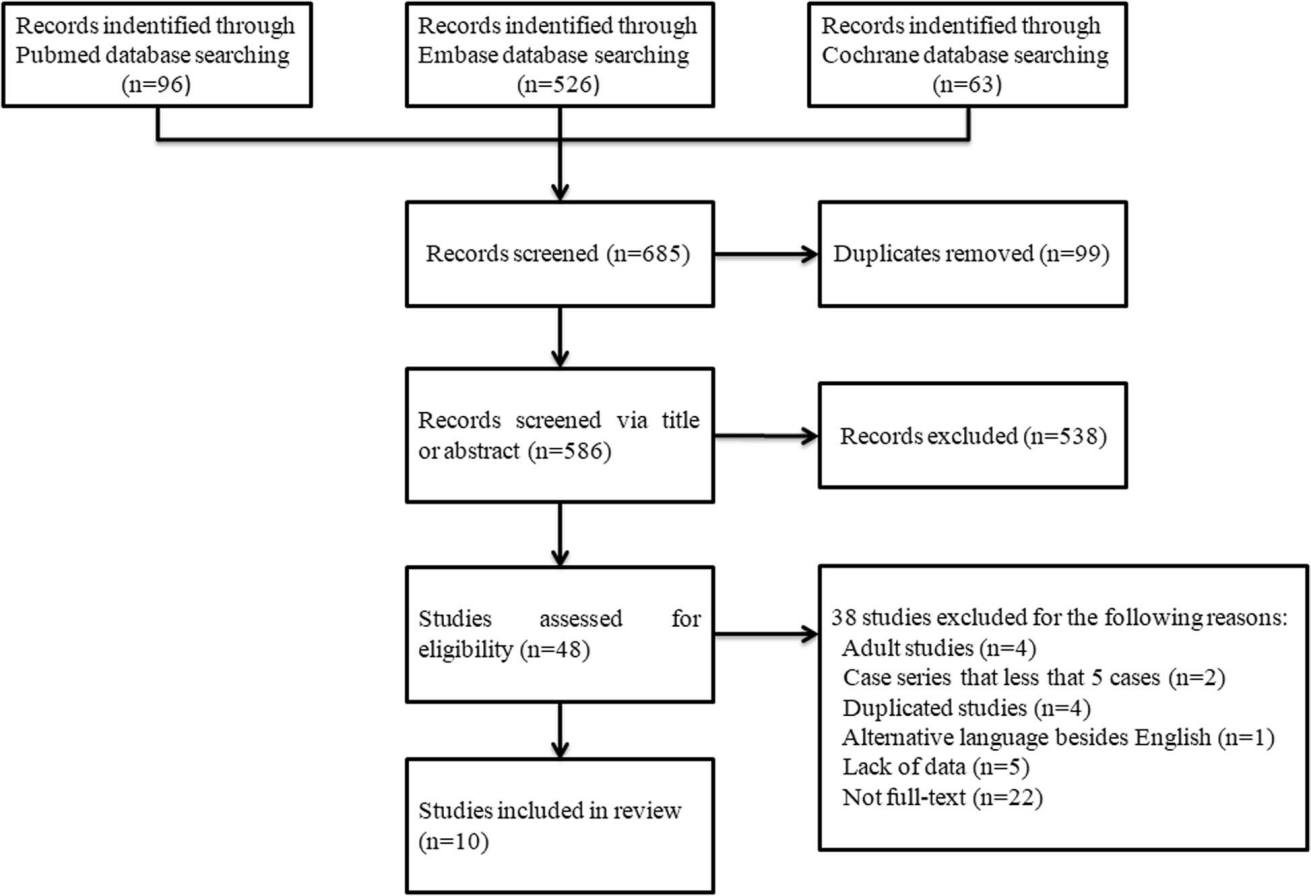
Flow chart of systematic review

Details of the search strategy are summarized in Fig. 1. A total of 685 citations were identified through PubMed, EMBASE and Cochrane library, of which 637 were excluded, based on the title or abstract. Forty-eight citations were evaluated in more details. Of these, thirty-eight were excluded for various reasons (Fig. 1), leaving 10 articles including a total of 455 patients (*n* = 216 CD, *n* = 239 UC/IBD-U) [22–31]. All studies focused on both CD and UC/IBD-U. Seven studies reported clinical remission rates [22–25, 29–31]. Four studies reported clinical response rates [23, 25, 29, 30]. Six studies reported CS-free remission rates [22–25, 28, 31] and 3 reported mucosal healing [24, 27, 29]. Nine studies reported safety outcomes for CD or UC/IBD-U combined, rather than by separate indication [22–26, 28–31]. Characteristics of the included studies are listed in Table 1, and patient demographics were showed in Table 2. These studies were mostly reported by institutions from the USA and differed with respect to patients’ age, number of patients included, concomitant treatment, vedolizumab dose, duration of treatment and follow-up, and definition of outcomes. Most patients received 300 mg vedolizumab, and others received 3.6–10.3 mg/kg vedolizumab.

**Table 2:** Baseline characteristics of the patients included in systematic review

Study ID	IBD type	Number of patients	Age years at vedolizumab initiation: median [range]/ Mean ± SD	Baseline severity		VDZ dose	Proportion of patients anti-TNF experienced	Previous medication	Concomitant medication	Disease behavior^a^
				**PCDAI median [range]/ Mean ± SD**	**PUCAI median [range]/pMS score median [range] / Mean ± SD**					
Singh[2016] [22]	CD/UC	52	14.9(7–17)	32.5 (17.5–45)	30 (IQR 10–55)	300 mg(*n* = 39); 6 mg/kg(*n* = 11); 5 mg/kg(*n* = 2)	47/52	IFX^b^ ADA^b^ CZP^b^	AZA/6-MP/MTX:15/52 CS:29/52	NR
Conrad[2016] [23]	CD/UC/IBD-U	21	13–18 (*n* = 15); 19–21 (*n* = 6)	25.0 (IQR 17.5–38.1)	30.0 (IQR 20.0–35.0)	300 mg	21/21	IFX:20/21 ADA:13/21 CZP:2/21 GLM:1/21	AZA/6-MP:1/21 MTX:8/21 CS:15/21	B1 9/16 B2 3/16 B3 2/16 B2&B3 2/16 P 8/16
Ledder[2017] [24]	CD/UC/IBD-U	64	10.7 ± 3.6	37.5 (IQR 24–61)	45 (IQR 30–65)	300 mg(*n* = 52); 150-250 mg(3.6–10.3 mg/kg)(*n* = 12)	64/64	Anti-TNF-α^c^ TAC:4/64 THA:1/64	AZA/6-MP/MTX:21/64 CS:41/64	B1 17/23 B2 5/23 B3 1/23 P 4/23 G 15/23
Schneider[2018] [25]	CD/UC/IBD-U	12	15(8–17)	Median:47.5	Median:70	6 mg/kg(Max 300 mg)	12/12	IFX:12/12 ADA:11/12 GLM:1/12	CS:8/11 AZA:3/11 5-ASA:2/11	NR
Olbjørn[2020] [26]	CD/UC	8	17(14–17.5)	68.75(IQR 65–76.25)	67.5(IQR 55.63–73.75)	300 mg	8/8	IFX:8/8 CS:8/8 AZA:3/8 MTX:3/8 5-ASA:5/8	IFX:8/8	B1 2/4 B3 2/4 P 1/4
Jossen[2020] [27]	CD/UC	68	16.4(IQR 13.3–18.2)	Anti-TNF naïve:26.2 (IQR 19.4–35.6) Anti-TNF exposed: 35 (IQR 25–57.5)	pMS: Anti-TNF naïve: 3.5 (2–5) pMS: Anti-TNF exposed: 6 (3–6.5)	≥ 30 kg:300 mg 19-29 kg:6-10 mg/kg	36/68	NR	CS:44/68	B1 29/33 B2 3/33 B3 1/33 P 10/33
Dolinger[2021] [28]	CD/UC/IBD-U	13	15.9 (IQR 13.5–16.9)	58.75 (IQR 31.25–58.75)	pMS:4 (IQR 0–7)	300 mg	13/13	Anti-TNF-α^c^	NR	B1 6/7 B2 1/7
Fabiszewska[2021] [29]	CD/UC	16	6.5(2.2–16.5)	34.4 ± 1.9	26 ± 6	150 mg or 300 mg depending on patient’s weight	15/16	IFX:15/16 ADA:9/16 Other biologics:4/16 CS:16/16 BUD:8/16 5-ASA:15/16 AZA:14/16 CsA:10/16	During induction phase: AZA:5/16 MTX:4/16 CS:8/16 During maintenance phase: AZA:4/14 MTX:4/14 CS:6/14	NR
Garcia-Romero [2021] [30]	CD/UC	42	12.6(IQR 8.9–14.0)	36 (IQR 24–60)	47 (IQR 25–65)	< 40 kg: 6 mg/kg (*n* = 20) 300 mg (*n* = 22)	42/42	IFX:40/42 ADA:26/42 ADA + IFX:24/42 Oral CS:39/42 IV CS:28/42 AZA:40/42 6-MP:4/42 MTX:12/42 CsA:5/42 5-ASA:34/42	CS:22/42 AZA:19/42 6-MP:1/42 MTX:6/42 5-ASA:22/42 TAC:2/22	B1 13/14 B2 0/14 B3 1/14 G 5/14
Hajjat[2021] [31]	CD/UC/IBD-U	159	14.5 ± 2.4	27.5 (IQR 15–40)	50 (IQR 35–65)	At VED start: 6.0 ± 1.8 mg/kg; At VED end: 5.2 ± 1.9 mg/kg	136/159	IFX:132/159 ADA:66/159 GLM:2/159 CZP:14/159 UST:5/159 CS:105/159 AZA:16/159 6-MP:48/159 MTX:82/159 5-ASA:78/159 SUL:19/159 RT:72/159	CS:101/159 AZA: 9/159 MTX: 46/159	B1 44/78 B2 17/78 B3 6/78 B2&B3 11/78

^a^Disease behavior classification was according to Paris classification

^b^The study did not provide the exact number of using IFX/ADA/CZP before vedolizumab therapy

^c^The study did not specify the kinds of anti-TNF-α agents

Abbreviations: *IBD* inflammatory bowel disease, *CD* Crohn’s disease, *UC* ulcerative disease, *IBD-U* inflammatory bowel disease unclassified, *SD* standard deviation, *IQR* interquartile range, *PCDAI* Pediatric Crohn’s Disease Activity Index, *PUCAI* Pediatric Ulcerative Disease Activity Index, *VDZ* vedolizumab, *pMS* partial Mayo score, *TNF* tumour necrosis factor, *CS* corticosteroids, *BUD* budesonide, *AZA* azathioprine, *MP* 6-mercaptopurine, *MTX* methrotrexate, *CsA* cyclosporine, *THA* thalidomide, *IFX* infliximab, *ADA* adalimumab, *GLM* golimumab, *CZP* certolizumab, *UST* ustekimumab, *5-ASA,5* aminosalicylic acid, *SUL* sulfasalazine, *RT* rectal therapy, *TAC* tacrolimus, *B1* Nonstricturing, *B2* Stricturing, *B3* Penetrating, *P* Perianal, *NR* not reported

### Primary outcome

#### Clinical remission

In CD patients, the short-term clinical remission rate at 2 weeks of therapy ranged from 0 to 27% [two studies] [22, 25], and at 6 weeks of treatment ranged from 0 to 35% [four studies] [22–25]. For maintenance therapy, the remission rates were ranging from 17 to 42% at 14 weeks [six studies] [22–25, 29, 30], 24% to 39% at 22 weeks [three studies] [22–24], 44% at 24 weeks [one study] [31], 31% to73% at 30 weeks [two studies] [22, 30], and 25% to 49% at 1 year [three studies] [24, 30, 31]. In UC/IBD-U patients, the short-term clinical remission rate at 2 weeks of therapy ranged from 40 to 41% [two studies] [22, 25], and at 6 weeks of treatment ranged from 20 to 64% [four studies] [22–25]. During maintenance treatment, the remission rates were ranging from 20 to 77% at 14 weeks [six studies] [22–25, 29, 30], 40% to 71% at 22 weeks [three studies] [22–24], 53% at 24 weeks [one study] [31], 65% to 75% at 30 weeks [two studies] [22, 30], and 41% to 60% at 1 year [three studies] [24, 30, 31].

### Secondary outcomes

#### CS-free clinical remission

The short-term CS-free clinical remission rates were 7% for CD [one study] [23] and 0% for UC/IBD-U [one study] [23] at 6 weeks respectively. For maintenance treatment, CS-free clinical remission rates in CD patients ranged from 0 to 19% at 14 weeks [three studies] [23–25], 13% to 33% at 22 weeks [three studies] [22–24], 41% at 24 weeks [one study] [31], 71% at 26 weeks [one study] [28], 0% at 38 weeks [one study] [25], and 45% at 1 year [one study] [31]. In UC patients, CS-free clinical remission rates were ranging from 20 to 44% at 14 weeks [three studies] [23–25], 40% to 71% at 22 weeks [three studies] [22–24], 49% at 24 weeks [one study] [31], 78% at 26 weeks [one study] [28], 80% at 38 weeks [one study] [25], and 41% at 1 year [one study] [31].

#### Clinical response

In CD patients, clinical response rates were 33% at 6 weeks [one study] [23], ranging from 33 to 75% at 14 weeks [four studies] [23, 25, 29, 30], 60% at 22 weeks [one study] [23], 46% at 30 weeks [one study] [30], and 50% at 1 year [one study] [30]. In UC/IBD-U patients, clinical response rates were 20% at 2 weeks [one study] [25], 25% at 6 weeks [one study] [23], 50% to 75% at 14 weeks [three studies] [23, 29, 30], 50% at 22 weeks [one study] [23], 78% at 30 weeks [one study] [30], and 71% at 1 year [one study] [30].

The pooled results for clinical remission rates, CS-free clinical remission, and response rates were presented in Table 3.

**Table 3:** Efficacy of Vedolizumab on pediatric inflammatory bowel disease

IBD type	Outcome measures	Number of studies	Overall[percentage; 95% CI]
**CD**	**Remission**		
	2 weeks	2	8/36[22;11–42]
	6 weeks	4	19/75[25;17–37]
	12 weeks	1	26/71[37;25–48]
	14 weeks	6	25/85[28;18–37]
	22 weeks	3	17/53[32;19–44]
	24 weeks	1	32/73[44;32–55]
	30 weeks	2	12/24[52;10–93]
	1 year	3	43/92[46;36–57]
	**Response**		
	6 weeks	1	5/15[33;9–57]
	14 weeks	4	20/39[52;37–67]
	22 weeks	1	9/15[60;35–85]
	30 weeks	1	6/13[46;19–73]
	1 year	1	5/10[50;19–81]
	**CS-free clinical remission**		
	6 weeks	1	1/15[7;-6–19]
	12 weeks	1	24/71[34;23–45]
	14 weeks	3	5/37[14;3–29]
	22 weeks	3	12/47[26;11–36]
	24 weeks	1	30/73[41;30–52]
	26 weeks	1	5/7[71;38–105]
	38 weeks	1	0/6[0;NA]
	1 year	1	35/78[45;34–56]
**UC/IBD-U**	**Remission**		
	2 weeks	2	11/27[41;22–59]
	6 weeks	4	25/70[36;10–57]
	12 weeks	1	37/79[47;36–58]
	14 weeks	6	52/101[48;31–65]
	22 weeks	3	24/45[53;36–73]
	24 weeks	1	42/79[53;42–64]
	30 weeks	2	21/31[68;52–84]
	1 year	3	50/112[45;35–54]
	**Response**		
	2 weeks	1	1/5[20;–15–55]
	6 weeks	1	1/4[25;–17–67]
	14 weeks	3	30/44[69;53–84]
	22 weeks	1	2/4[50;1–99]
	30 weeks	1	18/23[78;61–95]
	1 year	1	15/21[71;52–91]
	**CS-free clinical remission**		
	6 weeks	1	0/5[0;NA]
	12 weeks	1	35/79[44;33–55]
	14 weeks	3	17/44[39;17–51]
	22 weeks	3	26/45[58;44–73]
	24 weeks	1	39/79[49;38–60]
	26 weeks	1	7/9[78;51–105]
	38 weeks	1	4/5[80;45–115]
	1 year	1	33/81[41;30–51]

Abbreviations: *IBD*, inflammatory bowel disease, *CD* Crohn’s disease, *UC* ulcerative disease, *IBD-U* inflammatory bowel disease unspecified, *CS* corticosteroids

#### Mucosal healing

Three studies investigated mucosal healing, but one study did not draw clear conclusion due to the small sample size (*n* = 8) [29]. Another two studies (87 patients in total) reported mucosal healing results [24, 27]. Mucosal healing was found in 17%-39% of CD (*n* = 39) and 15%-34% of UC/IBD-U (*n* = 48) respectively, with various evaluation time. Details of number of patients assessed and evaluation time were presented in Table 4.

**Table 4:** Summary of mucosal healing rate among patients with Crohn’s disease or Ulcerative Colitis receiving vedolizumab

IBD type	Study	Patients with mucosal healing (n)	Patients assessed (n)	Rate (%)	Follow-up time(weeks):Median[IQR]
**CD**					
	Ledder (2017) [24]	1	6	17	24[14–38]
	Jossen (2020) [27]	13	33	39	49[32–73]
**UC/IBD-U**					
	Ledder (2017) [24]	2	13	15	24[14–36]
	Jossen (2020) [27]	12	35	34	49[32–73]

Abbreviations: *IBD*, inflammatory bowel disease, *CD* Crohn’s disease, *UC* ulcerative disease, *IBD-U* inflammatory bowel disease unspecified

#### Safety

Nine studies (*n* = 390) reported safety outcomes [22–26, 28–31]. Among them, one reported elevated transaminases and eczema which were considered unrelated to vedolizumab [26]; one reported upper respiratory tract infection which was uncertain to be related to vedolizumab [29];two studies did not report the quote of patients experiencing AEs, but only reported the total number of total AE registered [23, 30]. Serious AE rates were reported in 9 studies with 6 studies reporting zero [22, 24, 26, 29–31] and the other 3 studies reporting 6.25%-38.1% [23, 25, 28]. The most common AEs were respiratory tract infection and nausea and vomiting, followed by headache. Details were showed in Table 5.

**Table 5:** Adverse events during vedolizumab therapy

Any adverse events	No Reported Occurrences	Serious adverse events ^d^	No Reported Occurrences
Overall	NR	Overall	10/173
Respiratory tract infection^a^	15	Dehydration/vomiting	4
Nausea and vomiting	14	Flare of disease^e^	3
Headache	11	Bowel-associated dermatosis–arthritis syndrome^fe^	1
Fatigue	8	Synovitis, acne, pustulosis, hyperostosis, osteitis^g^	1
Mild-nonurticarial-rash	7	Obstructing nephrolithiasis and pyonephritis^h^	1
Arthralgia/joint pain	6	Diverting ileostomy^i^	1
Dizziness	5	ColectomySynovitis, acne, pustulosis, hyperostosis, osteitis^f^	11
Skin infections	2	Severe systemic allergic reactionObstructing nephrolithiasis and pyonephritis^g^	11
Dermatitis and rhinitis	2	Septic arthritis^j^Diverting ileostomy^h^	11
Erythema nodosum	2	Deep vein thrombosis^k^Colectomy	11
Allergic reaction^b^	2	Severe systemic allergic reaction	1
Otitis externa	1	Septic arthritis^i^	1
Periorbital oedema	1	Deep vein thrombosis^j^	1
Intractable itch	1		
New perianal disease	1		
Septic arthritis	1		
Deep vein thrombosis	1		
Cholangitis^c^			
Isolated cases of paraesthesia	1		
Alopecia	1		
Anaemia	1		
Herpes zoster	1		
Impetigo	1		

Abbreviations: *NR* no exact number reported (Conrad et al. (2016) [23]  and Garcia-Romero et al. (2021) [30] didn’t report number of patients who had adverse events)

^a^includes upper respiratory tract infection, nasopharyngitis, sinusitis

^b^includes mild shortness of breath and general systemic allergic reaction with dyspnoea

^c^The patient had a history of primary sclerosing cholangitis and had ascending cholangitis while on vedolizumab therapy

^d^includes requiring hospitalization or vedolizumab discontinued

^e^3 patients developed new extraintestinal manifestations of IBD, of which 2 subjects had new onset erythema nodosum, and 1 subject developed bowel-associated dermatosis–arthritis syndrome

^f^The subject who had diverting ileostomy due to severe perianal disease, developed bowel-associated dermatosis–arthritis syndrome and was treated with antibiotics and corticosteroids with subsequent resolution of symptoms and continued on vedolizumab without further recurrence of these manifestations

^g^The subject who initially had erythema nodosum, later developed synovitis, acne, pustulosis, hyperostosis, and osteitis syndrome, characterized by dermatologic and osteoarticular findings without clear etiology that has been associated with IBD in previous case reports

^h^The subject with CD, who had a history of recurrent acute kidney injury due to hypovolemia with disease flares, developed obstructing nephrolithiasis with associated pyonephritis, then underwent drainage and ureteral stent placement as well as intravenous antibiotic treatment, and was continued on vedolizumab achieving remission without further kidney involvement

^i^The subject with CD, who had worsening symptoms and distal colonic inflammation, required a diverting ileostomy

^j^The subject with UC treated with the combination of vedolizumab 300 mg every 8 weeks and tofacitinib 10 mg twice daily, in addition to prednisone 30 mg daily, developed septic arthritis of the right knee 2 months after dual therapy initiation, requiring inpatient hospitalization with incision and drainage and a prolonged course of intravenous antibiotic therapy

^k^The subject above subsequently developed a deep vein thrombosis in the right leg 5 months after dual therapy initiation

### Study quality

A 20-item validated quality appraisal tool for case series were used for quality assessment. The median of quality score was 17 (range 13–18), with only one study quality score less than 14[26].This study was case series which only safety data were involved in our study, and did not affect the quality of the whole analysis. The grade of evidence was showed in Table S2.

## Discussion

The results of this systematic review showed that most of the pediatric data on the effectiveness and safety of vedolizumab for the treatment of IBD were descriptive and the evidence were inadequate, as all the studies included were case series without randomized controlled trails (RCTs).

Overall, we found 0%-35% of CD patients achieved clinical remission in short-term therapy, compared to that of 20%-64% in UC patients. During maintenance therapy, 17%-73% of CD patients and 20%-77% of UC/IBD-U patients achieved clinical remission. Approximately 33%-75% of CD patients and 20%-78% of UC/IBD-U patients had clinical response with quite small sample size. These findings suggested similar therapeutic response were obtained in CD and UC, which were not consistent with previously published studies in adults. Randomized controlled trials of GEMINI 1 and 2 found that compared with CD, the response and remission rates in UC were higher at both 6 weeks (47.1% and 16.9% vs. 31.4% and 14.5%) and 52 weeks (56.6% and 41.8% vs. 39.0% and 43.5%) [10, 11]. Canadian and Hungarian real-world cohorts also showed significantly greater clinical remission and response rate for UC compared with CD [34, 35]. However, opposite results reported by Dragoni et al., cohort in Italy showed better results for CD patients, with higher clinical response and remission rate compared with UC at 14 weeks (85% and 69% vs. 52% and 30%), 24 weeks (84% and 61% vs. 56% and 26%) and 52 weeks (59% and 45% vs. 25% and 20%) [36]. The difference in clinical response and remission rate could be attributed to quite small sample size and differences in patients baseline characteristics variability: the characteristic of patients involved varied in IBD phenotype, disease severity at vedolizumab initiation, disease duration.

Steroid-free remission, whether clinically or endoscopically is an important treatment goal for pediatric IBD [20, 37], as corticosteroids have potentially serious side effects associated with long term use including linear growth restriction, and osteopenia amongst many others [38]. In a meta-analysis on adult population, approximately one-quarter of CD or UC achieved CS-free clinical remission at 14 weeks, while 31% of CD and 42% of UC of that at 12 months [39]. Our study seemingly showed similar results. In our study, we found 0%-19% of CD and 20%-44% of UC/IBD-U patients achieved CS-free clinical remission at 14 weeks. Higher rate was identified for UC (40%-71%) compared with CD (13%-33%) at 22 weeks, and similar rate was found at 1 year [45%(CD) vs. 41%(UC)]. However, opposite results have also been reported. In a real-world study by Zingone et al., better results for CD were identified at any follow-up time [ie, between 8–12 weeks 53.6% vs.18.7% (UC); 30 weeks 56.5% vs. 25% (UC); 52 weeks 53.6% vs. 35.4%(UC)], as much more CS ongoing UC were initially involved [45.8% vs. 24.6%(CD)] [40].

Although mucosal healing is a critical IBD therapy goal associated with sustained clinical remission, it is too burdensome for children to frequently undergo endoscopy. Therefore, only two studies of small sample size reported mucosal healing rates of 17%-39% for CD and 15%-34% for UC/IBD-U with median follow-up time over 6 months. In addition, the definition of mucosal healing is still controversial. Most investigators agree that an endoscopic Mayo subscore of 0 for UC, and simple endoscopic score for Crohn’s disease (SES-CD) 0–2 for CD [20]. However, one study involved in our review defined mucosal healing more strictly, including a composite of both endoscopic (macroscopic) and histologic indices [27]. In adult population, mucosal healing rates are reported as 21.2–41.9% for CD and 15%-57.1% for UC regardless of patients’ baseline characteristics [34–36, 41].

It is discussed, whether a previous treatment with anti TNF might influence the outcome of treatment with vedolizumab. Two post hoc analyses from the GEMINI studies assessed the efficacy of vedolizumab in CD and UC based on previously anti‐TNF experienced patients [42, 43]. Results showed for CD, there were higher response and remission rates in patients who were anti-TNF-naïve compared with anti-TNF-experienced, and the advantages persisted to week 52 [42]. In UC patients, similar outcomes were found. Compared to placebo, patients naïve to anti‐TNF had higher rates of response than patients with anti‐TNF failure at week 6, whereas during maintenance therapy, there were no significant difference with placebo in both groups and between the two groups [43]. In contrast, however, some real-world clinical studies indicated that there was no impact of previous anti‐TNF exposure on response or maintenance of remission though the sample size of TNF–naïve patients were small [34, 44]. Chaparro et al. found the remission rates of patients who were anti-TNF naïve, with failure to 1 anti‐TNF and failure to > 1 anti‐TNF at week 14 were 57.6%, 51.2% and 44% respectively [44]. And a study by Kotze et al. demonstrated that previous failure to anti‐TNF agents was not associated with the efficacy of vedolizumab [34]. More interestingly, a study by Mader showed previous treatment with anti-TNF agents was associated with a significantly lower efficacy of VDZ in UC but not in CD patients [45]. This might be attributed to longer disease duration for anti-TNF-experienced UC patients. In pediatric population, Jossen et al. found higher rates of both endoscopic and histologic remission in anti-TNF-naïve patients compared to those who were anti-TNF-experienced (66% vs. 42%, 52% vs 33%, respectively) [27]. However, authors admitted these anti-TNF-naïve patients had slightly less severe disease at baseline compared with the anti-TNF-experienced patients [wPCDAI 26.2(19.4–35.6) vs. 35(25–57.5); pMayo 3.5(2–5) vs. (3–6.5)]. Therefore, this question deserves further investigation to determine whether the differences are due to true biological effects of anti-TNF exposure or the severity and duration of the disease reflected in patients who started using vedolizumab.

With respect to safety, phase 2 and 3 trials showed a favorable safety profile of vedolizumab, with similar AEs incidence rate compared with placebo [46, 47]. Safety data from real-world cohort studies reported the total AE incidence rate was 23.6%, with infectious complication rate 7.8% [47]. In pre-marketing clinical trials, the most frequently reported AEs were respiratory tract infection [21.2/100 person-years (PYs)] and abdominal pain (12.1/100 PYs) [46, 47]. And real-world data showed respiratory tract infection (3.6%) and arthralgia (3.1%) were most common AEs [47]. Our findings were basically consistent with those from adult populations. The most prevalent AEs were respiratory tract infection and nausea and vomiting. Nevertheless, one study reported by Conrad et al. reported 38% (8/21) experienced 12 serious adverse events that required hospitalization [23].

There are several limitations in our review. Initially, there was a significant heterogeneity in study design, including the threshold criteria of patients involved and definitions of remission, response and mucosal healing. Most studies used Pediatric Crohn’s Disease Activity Index (PCDAI) or Pediatric Ulcerative Disease Activity Index (PUCAI), but weighted Pediatric Crohn’s Disease Activity Index (wPCDAI), short Pediatric Crohn’s Disease Activity Index (shPCDAI) or partial Mayo score were also used. As to mucosal healing, endoscopic assessment alone was agreed by majority of investigators, but Jossen et al. also evaluated histological changes [27]. Moreover, all the studies included were case series, some reported the data prospectively while the others used a retrospective approach, which may result in significant differences in clinical decision. In addition, there was no placebo-controlled trial with a standard protocol, which meant the effectiveness was not necessarily attributed to the intervention. The recurrent nature of CD additionally weakens the assessment of causal relationships between interventions and outcomes. Nevertheless, vedolizumab for pediatric patients is usually applied to patients with severe disease or those who are refractory to conventional therapies, which are unlikely to have spontaneous relief.

In spite of the above-mentioned shortcomings, we performed a comprehensive literature search. Although no RCTs were included, case series of vedolizumab therapy seemed to represent ‘real-world’ experience of pediatric population in different areas and medical centers and provide a deeper understanding of vedolizumab in heterogenous and more complex patient populations. Besides, the role of case series evidence in systematic reviews of health care interventions is especially suitable for reviews of rapidly developing pharmacological interventions and supporting evidence on safety, when case series are usually the only available clinical evidence [48].

## Conclusions

Based on low-quality evidence provided by case series, approximately one-third and one-half of patients for CD and UC/IBD-U respectively, achieved remission within 22 weeks with favorable safety profile, and about half of patients achieved remission at 1 year with reasonable safety profile. Long-term benefit profile data and more robust evidence are still needed.

## Supplementary Information

**Additional file 1: Table S1. **Search strategy in databases.** Table S2. **Methodological quality of case series.

## Data Availability

All data used during the study are available from the corresponding author by request.

## Abbreviations

AEs: Adverse events
CD: Crohn’s disease
CI: Confidence interval
CS: Corticosteroid
ECCO: European Crohn's and Colitis Organization
ESPGHAN: European Society for Pediatric Gastroenterology Hepatology and Nutrition
IBD: Inflammatory bowel disease
IBD-U: Inflammatory bowel disease-unspecified
IHE: Institute of Health Economics
IQR: Interquartile range
MAdCAM-1: Mucosal addressing cellular adhesion molecule-1
PCDAI: Pediatric Crohn’s Disease Activity Index
PUCAI: Pediatric Ulcerative Disease Activity Index
RCTs: Randomized controlled trials
SD: Standard deviation
SES-CD: Simple endoscopic score for Crohn’s disease
shPCDAI: Short Pediatric Crohn’s Disease Activity Index
UC: Ulcerative colitis
wPCDAI: Weighted Pediatric Crohn’s Disease Activity Index
