# Supplementary material for: Efficacy and safety of vedolizumab for pediatrics with inflammatory bowel disease: a systematic review
Source: BMC Pediatr. 2022 Apr 4;22:175. doi: 10.1186/s12887-022-03229-x (PMC8978350; doi:10.1186/s12887-022-03229-x)
Supplement: Supplementary file 1 — Additional file 1: Table S1. Search strategy in databases. Table S2. Methodological quality of case series. [file 12887_2022_3229_MOESM1_ESM.doc]

**Table S1 Search strategy in databases**

| PubMed database search strategy | | | |
| --- | --- | --- | --- |
| Search number | Query | Search Details | Results |
| 12 | ((#3) AND (#8)) AND (#11) | ("Inflammatory Bowel Diseases"[MeSH Terms] OR ("crohn*"[Title/Abstract] OR "ulcerative colitis"[Title/Abstract] OR "IBD"[Title/Abstract] OR "inflammatory bowel disease*"[Title/Abstract])) AND ("Adolescent"[MeSH Terms] OR "Child"[MeSH Terms] OR "Pediatrics"[MeSH Terms] OR ("minors"[Title/Abstract] OR "boy"[Title/Abstract] OR "girl*"[Title/Abstract] OR "kid"[Title/Abstract] OR "child*"[Title/Abstract] OR "schoolchild*"[Title/Abstract] OR "adolescen*"[Title/Abstract] OR "juvenil*"[Title/Abstract] OR "youth*"[Title/Abstract] OR "teen*"[Title/Abstract] OR "preteen*"[Title/Abstract] OR "underage*"[Title/Abstract] OR "under age*"[Title/Abstract] OR "pubescen*"[Title/Abstract] OR "paediatric*"[Title/Abstract])) AND ("vedolizumab"[Supplementary Concept] OR ("Entyvio"[Title/Abstract] OR "MLN0002"[Title/Abstract] OR "MLN02"[Title/Abstract] OR "MLN-0002"[Title/Abstract] OR "MLN-02"[Title/Abstract])) | 96 |
| 11 | (#9) OR (#10) | "vedolizumab"[Supplementary Concept] OR "Entyvio"[Title/Abstract] OR "MLN0002"[Title/Abstract] OR "MLN02"[Title/Abstract] OR "MLN-0002"[Title/Abstract] OR "MLN-02"[Title/Abstract] | 766 |
| 10 | ((((Entyvio[Title/Abstract]) OR (MLN0002[Title/Abstract])) OR (MLN02[Title/Abstract])) OR (MLN-0002[Title/Abstract])) OR (MLN-02[Title/Abstract]) | "Entyvio"[Title/Abstract] OR "MLN0002"[Title/Abstract] OR "MLN02"[Title/Abstract] OR "MLN-0002"[Title/Abstract] OR "MLN-02"[Title/Abstract] | 56 |
| 9 | "vedolizumab" [Supplementary Concept] | "vedolizumab"[Supplementary Concept] | 728 |
| 8 | (((#4) OR (#5)) OR (#6)) OR (#7) | "Adolescent"[MeSH Terms] OR "Child"[MeSH Terms] OR "Pediatrics"[MeSH Terms] OR "minors"[Title/Abstract] OR "boy"[Title/Abstract] OR "girl*"[Title/Abstract] OR "kid"[Title/Abstract] OR "child*"[Title/Abstract] OR "schoolchild*"[Title/Abstract] OR "adolescen*"[Title/Abstract] OR "juvenil*"[Title/Abstract] OR "youth*"[Title/Abstract] OR "teen*"[Title/Abstract] OR "preteen*"[Title/Abstract] OR "underage*"[Title/Abstract] OR "under age*"[Title/Abstract] OR "pubescen*"[Title/Abstract] OR "paediatric*"[Title/Abstract] | 3,844,569 |
| 7 | ((((((((((((((minors[Title/Abstract]) OR (boy*[Title/Abstract])) OR (girl*[Title/Abstract])) OR (kid*[Title/Abstract])) OR (child*[Title/Abstract])) OR (schoolchild*[Title/Abstract])) OR (adolescen*[Title/Abstract])) OR (juvenil*[Title/Abstract])) OR (youth*[Title/Abstract])) OR (teen*[Title/Abstract])) OR (preteen*[Title/Abstract])) OR (underage*[Title/Abstract])) OR (under age*[Title/Abstract])) OR (pubescen*[Title/Abstract])) OR (paediatric*[Title/Abstract]) | "minors"[Title/Abstract] OR "boy"[Title/Abstract] OR "girl*"[Title/Abstract] OR "kid"[Title/Abstract] OR "child*"[Title/Abstract] OR "schoolchild*"[Title/Abstract] OR "adolescen*"[Title/Abstract] OR "juvenil*"[Title/Abstract] OR "youth*"[Title/Abstract] OR "teen*"[Title/Abstract] OR "preteen*"[Title/Abstract] OR "underage*"[Title/Abstract] OR "under age*"[Title/Abstract] OR "pubescen*"[Title/Abstract] OR "paediatric*"[Title/Abstract] | 1,971,703 |
| 6 | "Pediatrics"[Mesh] | "Pediatrics"[MeSH Terms] | 61,624 |
| 5 | "Child"[Mesh] | "Child"[MeSH Terms] | 2.031,435 |
| 4 | "Adolescent"[Mesh] | "Adolescent"[MeSH Terms] | 2,143,021 |
| 3 | #1 OR #2 | "Inflammatory Bowel Diseases"[MeSH Terms] OR "crohn*"[Title/Abstract] OR "ulcerative colitis"[Title/Abstract] OR "IBD"[Title/Abstract] OR "inflammatory bowel disease*"[Title/Abstract] | 125,947 |
| 2 | (((Crohn*[Title/Abstract]) OR (Ulcerative colitis[Title/Abstract])) OR (IBD[Title/Abstract])) OR (Inflammatory bowel disease*[Title/Abstract]) | "crohn*"[Title/Abstract] OR "ulcerative colitis"[Title/Abstract] OR "IBD"[Title/Abstract] OR "inflammatory bowel disease*"[Title/Abstract] | 112,741 |
| 1 | "Inflammatory Bowel Diseases"[Mesh] | "Inflammatory Bowel Diseases"[MeSH Terms] | 88,222 |

| **EMBASE database search strategy** | | |
| --- | --- | --- |
| No. | Query | Results |
| #15 | #6 AND #11 AND #14 | 526 |
| #14 | #12 OR #13 | 5,421 |
| #13 | entyvio:ab,ti OR 'ldp 02':ab,ti OR ldp02:ab,ti OR 'mln 0002':ab,ti OR 'mln 02':ab,ti OR 'mln 02 antibody':ab,ti OR 'mln 02 monoclonal antibody':ab,ti OR mln0002:ab,ti OR mln02:ab,ti OR 'mln02 antibody':ab,ti OR 'mln02 monoclonal antibody':ab,ti OR 'monoclonal antibody ldp 02':ab,ti OR 'monoclonal antibody mln 02':ti,ab,kw | 95 |
| #12 | 'vedolizumab'/exp | 5,415 |
| #11 | #7 OR #8 OR #9 OR #10 | 5,395,019 |
| #10 | minors:ab,ti OR boy*:ab,ti OR girl*:ab,ti OR kid*:ab,ti OR child*:ab,ti OR schoolchild*:ab,ti OR adolescen*:ab,ti OR juvenil*:ab,ti OR youth*:ab,ti OR teen*:ab,ti OR preteen*:ab,ti OR underage*:ab,ti OR 'under age*':ti,ab,kw OR pubescen*:ab,ti OR paediatric*:ab,ti OR pediatric*:ab,ti | 3,399,263 |
| #9 | 'pediatrics'/exp | 126,747 |
| #8 | 'child'/exp | 3,108,384 |
| #7 | 'adolescent'/exp | 1,785,005 |
| #6 | #1 OR #2 OR #3 OR #4 OR #5 | 214,671 |
| #5 | 'inflammatory bowel disease*':ab,ti | 89,173 |
| #4 | ibd:ab,ti | 60,227 |
| #3 | 'ulcerative colitis':ab,ti | 70,003 |
| #2 | crohn*:ab,ti | 87,683 |
| #1 | 'inflammatory bowel disease'/exp | 176,795 |
| **Cochrane database search strategy** | | |
| No. | Query | Results |
| #19 | #6 AND #15 AND #18 | 63 |
| #18 | #16 OR #17 | 448 |
| #17 | (MLN-02):ti,ab,kw | 2 |
| #16 | (vedolizumab):ti,ab,kw OR (Entyvio):ti,ab,kw OR (MLN0002):ti,ab,kw OR (MLN02):ti,ab,kw OR (MLN-0002):ti,ab,kw | 447 |
| #15 | #7 OR #8 OR #9 OR #14 | 384,636 |
| #14 | #10 OR #11 OR #12 OR #13 | 384,494 |
| #13 | (underage*):ti,ab,kw OR (under age*):ti,ab kw OR (pubescen*):ti,ab,kw OR (paediatric*):ti,ab,kw OR (padiatric*):ti,ab,kw | 90660 |
| #12 | (adolescen*):ti,ab,kw OR (juvenil*):ti,ab,kw OR (youth*):ti,ab,kw OR (teen*):ti,ab,kw OR (preteen*):ti,ab,kw | 149,847 |
| #11 | (boy*):ti,ab,kw OR (girl*):ti,ab,kw OR (kid*):ti,ab,kw OR (child*):ti,ab,kw OR (schoolchild*):ti,ab,kw | 226,985 |
| #10 | (minors):ti,ab.kw | 265 |
| #9 | MeSH descriptor: [Pediatrics] explode all trees | 713 |
| #8 | MeSH descriptor: [Child] explode all trees | 59,662 |
| #7 | MeSH descriptor: [Adolescent] explode all trees | 108,631 |
| #6 | #1 OR #2 OR #3 OR #4 OR #5 | 11,076 |
| #5 | (inflammatory bowel disease*):ti,ab,kw | 3,862 |
| #4 | (IBD):ti,ab,kw | 1,986 |
| #3 | (Ulcerative colitis):ti,ab,kw | 5,268 |
| #2 | (Crohn*):ti,ab,kw | 5,151 |
| #1 | MeSH descriptor: [Inflammatory Bowel Diseases] explode all trees | 3,586 |

**Table S2 Methodological quality of case series**

|  |  | Singh^24^ | Conrad^25^ | Ledder^26^ | Schneider^27^ | Olbjørn^28^ | Jossen^29^ | Dolinger^30^ | Fabiszewska^31^ | Garcia-Romero^32^ | Hajjat^33^ |
| --- | --- | --- | --- | --- | --- | --- | --- | --- | --- | --- | --- |
| 1. Is the hypothesis/aim/ objective of the study clearly stated? | Yes  Unclear  No | Yes | Yes | Yes | Yes | Yes | Yes | Yes | Yes | Yes | Yes |
| 2. Are the characteristics of the participants included in the study described? | Yes  Partially reported  No | Yes | Yes | Yes | Yes | Yes | Yes | Yes | Yes | Yes | Yes |
| 3. Were the cases collected in more than one centre? | Yes  Unclear  No | Yes | No | Yes | Yes | No | No | No | No | Yes | Yes |
| 4. Are the eligibility criteria (i.e. inclusion and exclusion criteria) for entry into the study clearly stated? | Yes  Partially reported  No | Yes | Yes | Yes | Yes | Yes | Yes | Yes | Yes | Yes | Yes |
| 5. Were participants recruited consecutively? | Yes  Unclear  No | Yes | Yes | Yes | Yes | Yes | Yes | Yes | Yes | Yes | Yes |
| 6. Did participants enter the study at a similar point in the disease? | Yes  Unclear  No | No | Yes | Yes | Yes | Yes | No | Yes | No | No | No |
| 7. Was the intervention of interest clearly described? | Yes  Partially reported  No | Yes | Yes | Yes | Yes | Yes | Yes | Yes | Yes | Yes | Yes |
| 8. Were additional interventions (co-interventions) reported in the study? | Yes  Unclear  No | Yes | Yes | Yes | Yes | Yes | Yes | Yes | Yes | Yes | Yes |
| 9. Are the outcome measures established a priori? | Yes  Partially reported  No | Yes | Yes | Yes | Yes | Yes | Yes | Yes | Yes | Yes | Yes |
| 10. Were the relevant outcomes measured with appropriate objective and/or subjective methods? | Yes  Unclear  No | Yes | Yes | Yes | Yes | Un | Yes | Yes | Yes | Yes | Yes |
| 11. Were the relevant outcomes measured before and after the intervention? | Yes  Unclear  No | Yes | Yes | Yes | Yes | Un | Yes | Yes | Yes | Yes | Yes |
| 12. Were the statistical tests used to assess the relevant outcomes appropriate? | Yes  Unclear  No | Yes | Yes | Yes | Yes | Un | Yes | Yes | Yes | Yes | Yes |
| 13. Was the length of follow-up reported? | Yes  Unclear  No | Yes | Yes | Yes | Yes | Yes | Yes | Yes | Yes | Yes | Yes |
| 14. Was the loss to follow-up reported? | Yes  Unclear  No | Yes | Yes | Yes | Yes | Yes | Yes | Yes | Yes | Yes | Yes |
| 15. Does the study provide estimates of the random variability in the data analysis of relevant outcomes? | Yes  Partially reported  No | Yes | Yes | Yes | Yes | No | Yes | Yes | Yes | Yes | Yes |
| 16. Are the adverse events related with the intervention reported? | Yes  Partially reported  No | Yes | Yes | Yes | Yes | Yes | No | Yes | Yes | Yes | Yes |
| 17. Are the conclusions of the study supported by results? | Yes  Partially reported  No | Yes | Yes | Yes | Yes | Yes | Yes | Yes | Yes | Yes | Yes |
| 18. Are both competing interests and sources of support for the study reported? | Yes  Partially reported  No | Yes | Yes | Yes | Yes | Yes | Yes | Yes | Yes | Yes | Yes |
| 19. Was the study conducted prospectively? | Yes  Unclear  No | No | Yes | No | No | No | No | Yes | No | No | No |
| 20. Were the relevant outcomes assessed blinded to intervention status? | Yes  Unclear  No | No | No | No | No | No | No | No | No | No | No |
| Total score |  | 17 | 18 | 18 | 18 | 13 | 15 | 18 | 16 | 17 | 17 |
